# Self-rated health, physical activity, measures of physical functioning and mortality among older U.S. adults

**DOI:** 10.1093/geroni/igaf122.4005

**Published:** 2025-12-31

**Authors:** Peter Hart

**Affiliations:** Kinesmetrics, Tallahassee, Florida, United States

## Abstract

The objective of this study was to determine if different measures of physical functioning (PF) can predict mortality independent of self-rated health (SRH) and physical activity (PA). A baseline sample of 6,173 adults 65+ years of age were included from the 2001-2018 NHANES. A SRH variable was created with categories of excellent/very good, good, fair, and poor. PA status was based on participants reporting either no (inactive) or at least some (active) recreational PA. Seven different PF measures were used and included a 19-item total PF score (PFT), activities of daily living (ADL), instrumental activities of daily living (IADL), leisure and social activities (LSA), general physical activities (GPA), lower extremity mobility (LEM), and an IRT-derived total PF score (PFIRT). All PF measures were scored so larger values represented greater PF limitation. Seven Cox regression models were employed each with a different PF measure and adjusted for age, sex, race, income, SRH, PA, BMI, BSI, smoking, alcohol consumption, and comorbidities. A total of 2,103 deaths occurred during a median follow-up of 10.3 years. Risk of death decreased for 1st (HR = 0.70, 0.60-0.82), 2nd (HR = 0.77, 0.64-0.93), and 3rd (HR = 0.82, 0.72-0.94) PFIRT quartiles (reference: 4th), increased for poor (HR = 2.11, 1.51-2.97), fair (HR = 1.82, 1.54-2.15), and good (HR = 1.20, 1.07-1.35) SRH (reference: excellent/very good), and increased for inactive (HR = 1.27, 1.12-1.43) PA status (reference: active). All PF models saw similar results less LSA where LSA lost its predictive ability. These findings indicate that SRH, PA, and PF are robust independent predictors of all-cause mortality in older adults.

